# LTC_4_ synthase polymorphism modifies efficacy of botanical seed oil combination in asthma

**DOI:** 10.1186/2193-1801-3-661

**Published:** 2014-11-06

**Authors:** Shamsah Kazani, Jonathan P Arm, Joshua Boyce, Heng Chhay, Stefanie Dutile, Michael E Wechsler, Usha Govindarajulu, Priscilla Ivester, Hannah C Ainsworth, Susan Sergeant, Floyd H Chilton, Elliot Israel

**Affiliations:** Division of Pulmonary and Critical Care Medicine, Brigham and Women’s Hospital, 75 Francis Street, Boston, MA 02115 USA; Division of Rheumatology, Immunology and Allergy, Brigham and Women’s Hospital, Harvard Medical School, Boston, MA USA; Departments of Physiology/Pharmacology, Wake Forest University Health Sciences, Winston-Salem, NC USA; Departments of Biochemistry, Wake Forest University Health Sciences, Winston-Salem, NC USA; Wake Forest University Center for Botanical Lipids and Inflammatory Disease Prevention, Wake Forest University Health Sciences, Winston-Salem, NC USA

**Keywords:** Asthma, Borage oil, Echium oil, Leukotrienes, LTC_4_ synthase

## Abstract

Botanical seed oils reduce the generation of leukotrienes in patients with asthma.

Our objective was to determine the efficacy of a botanical seed oil combination against airflow obstruction in asthma, and to determine the pharmacogenomic effect of the leukotriene C_4_ synthase (LTC_4_S) polymorphism A-444C.

We conducted a randomized, double-blind, placebo-controlled, cross-over clinical trial in mild to moderate asthmatics to determine the change in FEV_1_ after 6 weeks of therapy with borage and echium seed oils versus corn oil placebo. We also examined the effect of the variant LTC_4_S -444C allele on the change in lung function.

We did not identify a difference in FEV_1_ in the study cohort as a whole (n = 28), nor in the group of A homozygotes. In the C allele carriers (n = 9), FEV_1_ improved by 3% after treatment with borage and echium seed oils and declined by 4% after placebo corn oil (p = 0.02). All 9 C allele carriers demonstrated an improvement in their FEV_1_ on active treatment compared to placebo as compared to only 7 out of 19 A allele homozygotes (p = 0.007). We observed transient differences in *ex vivo* leukotriene generation from circulating basophils and granulocytes. We did not observe significant differences in urinary LTE_4_ levels.

We conclude that compared to corn oil, a combination of borage and echium seed oils improves airflow obstruction in mild to moderate asthmatics who carry the variant allele in the LTC_4_S gene (A-444C). Botanical oil supplementation may have therapeutic potential in asthma if used in a personalized manner.

**Trial registration:** This trial was registered at http://www.clinicaltrials.gov as NCT00806442.

## Background

5-lipoxygenase (5-LO)-dependent oxidative metabolism of arachidonic acid (AA) leads to generation of leukotrienes (LTs), which are associated with airway inflammation in asthma (Peters-Golden and Henderson 2007). Studies have demonstrated that dietary supplementation with marine or botanical seed oils containing omega-3 and omega-6 polyunsaturated fatty acids (PUFAs) can decrease airway inflammation by reducing the generation of leukotrienes and pro-inflammatory cytokines, and attenuating neutrophil function (Barros et al. 2011; Chilton-Lopez et al. 1996; Lee et al. 1985).

**Figure 1:**
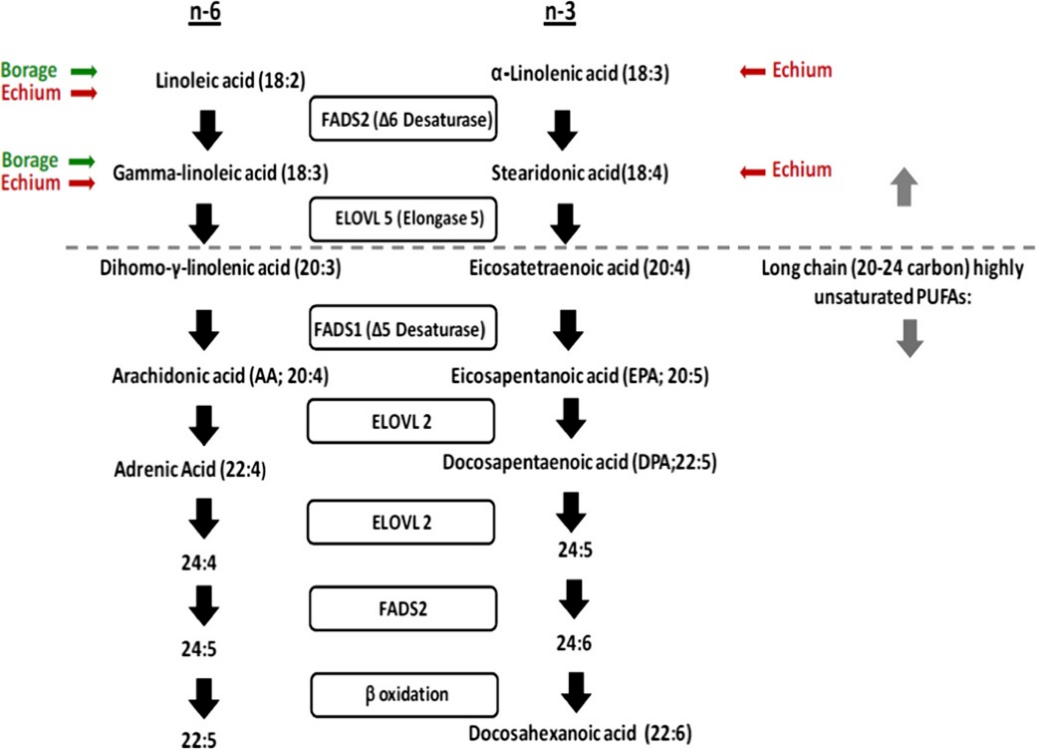
**Pathways for metabolism of omega-6 (left) and omega-3 (right) fatty acids in humans.** The synthesis of long chain PUFAs from the essential dietary medium chain PUFAs, α-linolenic acid (ω-3) and linoleic acid (ω-6). The fatty acids derived from borage oil (linoleic and gamma-linoleic acids, both ω-6) and echium (stearidonic, ω-3; linoleic and gamma-linoleic acids, both ω-6) would be expected to enter the pathways as indicated.

Seed oils from the Boraginaceae family of plants, including borage oil (*Borago officinalis*) and echium oil (*Echium plantagineum*) contain medium chain omega-6 and omega-3 PUFAs, including γ-linolenic acid (GLA;18:3,n-6), α-linolenic acid (ALA;18:3,n-3) and stearidonic acid (SDA;18:4,n-3) (Figure 1). GLA is efficiently converted by cells and tissues to dihomo γ-linolenic acid (DGLA) that competes with AA for substrate utilization by 5-LO, thus having anti-inflammatory potential in asthma. However, the conversion of ALA to long-chain omega-3 PUFAs such as eicosapentaenoic acid (EPA) and docosahexaenoic acid (DHA) (both known to reduce leukotrienes) is poor in humans, which is believed to be a result of the inefficiency of the initial rate-limiting step (Δ-6 desaturase, FADS2 gene) involved in long-chain PUFA biosynthesis. However SDA is downstream of Δ-6 desaturase and is up to five-fold more efficiently converted to EPA than ALA (James et al. 2003). Additionally, SDA has been demonstrated to block leukotriene generation from leukocytes *in vitro* (Guichardant et al. 1993). Further, our preliminary dose-titration study has suggested that a combination of 1.7 g/day of GLA and 0.8 g/day of SDA obtained from a mixture of borage and echium seed oils can significantly reduce the generation of leukotrienes from AA without impacting circulating AA levels (Arm et al. 2013). However, the physiologic effects of botanical seed oils on improvement in airflow obstruction in asthma are not known. Hence, we conducted a randomized, double-blind, placebo-controlled, cross-over clinical trial in mild to moderate asthmatics comparing the change in forced expiratory volume in one second (FEV_1_) after 6 weeks of therapy with the botanical oil combination.

The ability of inflammatory cells to generate leukotrienes is influenced by polymorphisms in genes involved in leukotriene synthesis (Sampson et al. 2000). Of particular interest is a common A to C single nucleotide polymorphism located 444 base pairs upstream of the gene encoding leukotriene C_4_ synthase (LTC_4_S A-444C, rs730012). The dominant variant C allele of the gene for LTC_4_ synthase has been associated with an increase in cysteinyl LT production, reduced lung function and increased effectiveness of LT receptor antagonists against bronchoconstriction in asthma (Sampson et al. 2000; Silverman et al. 1998; Tantisira and Drazen 2009). To our knowledge, no studies have examined whether the LTC_4_S variant allele has an impact on the efficacy of PUFA supplementation on physiologic outcomes associated with asthma. Hence we hypothesized that dietary supplementation with the botanical seed oil combination would improve airflow obstruction in asthmatics when compared to placebo, preferentially in those who carried the variant C allele at the LTC_4_S A-444C locus.

## Results

**Figure 2:**
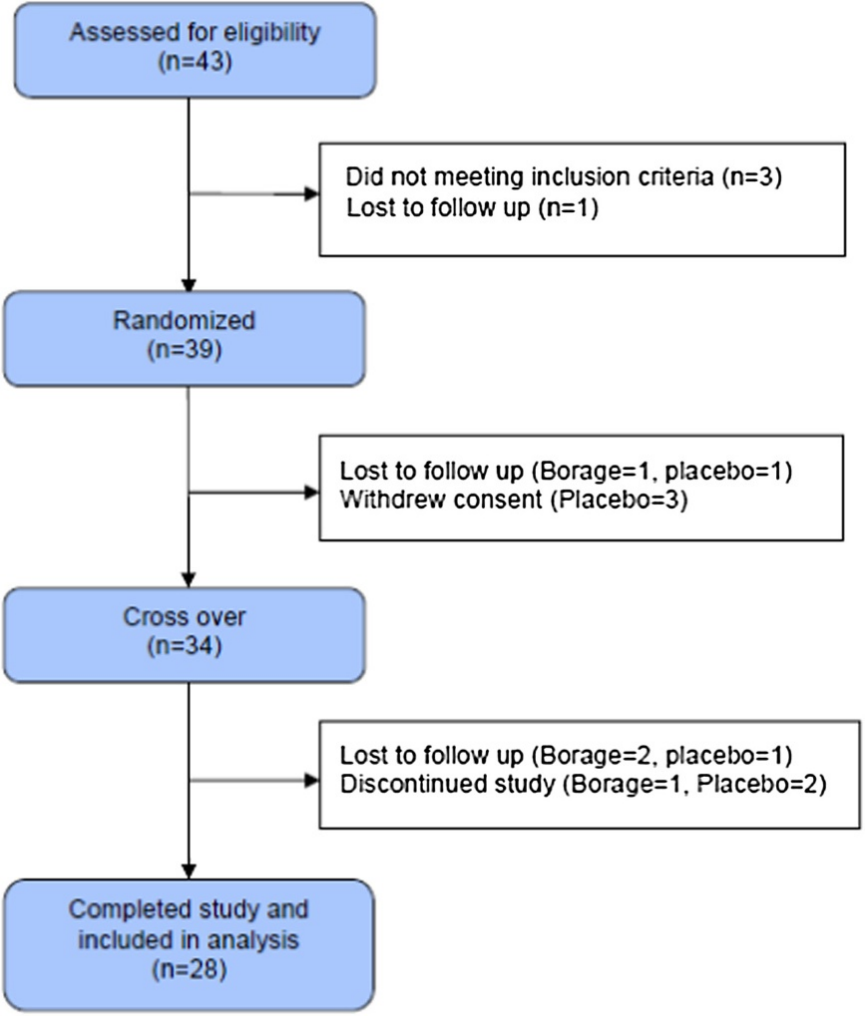
**Study enrollment and randomization flow sheet.**

We enrolled 43 participants with mild to moderate asthma and randomized 39, of which 28 completed the study (Figure 2). Nine participants were found to carry at least one copy of the variant C allele in the LTC_4_S promoter. There were no significant differences in baseline clinical characteristics between the two genotype groups (Table 1).

**Table 1:** **Participant’s characteristics and study results**

Characteristics and results	A homozygotes (AA)	C allele carriers (AC/CC)
N	19	9 (8 AC, 1 CC)
Age (years)^a^	37 ± 14 (20 – 60)	29 ± 8 (18 – 39)
Sex (female/male)	14/5	8/1
Race (Caucasian/other)	15/4	7/2
Hispanic (yes/no)	1/18	3/6
ICS dose (μg fluticasone)^a^	117 ± 250 (0 – 1000)	85 ± 128 (0 – 267)
Baseline rescue inhaler use (puffs/week)^a^	5 ± 6 (0 – 18)	7 ± 7 (0 – 20)
Baseline FEV_1_ (L)^a^	2.24 ± 0.51 (1.35 – 3.01)	2.31 ± 0.47 (1.75 – 3.26)
Baseline FEV_1_ (%)^a^	69 ± 12 (51 – 89)	69 ± 9 (55 – 84)
Change in FEV_1_ after placebo (%)	1 ± 9 (-21 – 18)	−4 ± 6 (-15 – 3)
Change in FEV_1_ after drug (%)^a^	1 ± 5 (-11 – 9)	3 ± 5 (-3 – 12)
Change in FEV_1_ after drug compared to placebo (%)^a*^	−1 ± 9 (-13 – 24)	7 ± 5 (1 – 17)

*p value for difference between group means =0.02.

^a^Mean ± standard deviation (minimum – maximum).

**Figure 3:**
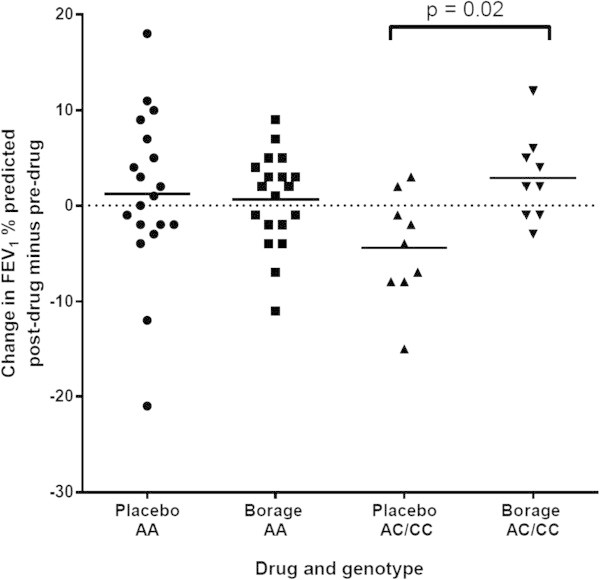
**Change in FEV**_**1**_ **% predicted in participants from both genotype groups after 6 weeks of therapy with drug and placebo.** Horizontal bars represent group means.

**Figure 4:**
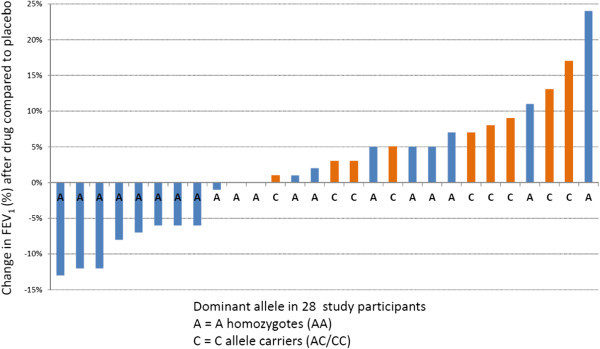
**Effect of 6 weeks of dietary supplementation with borage and echium seed oils on FEV**_**1**_ **compared to corn oil placebo in 28 mild to moderate asthmatics.** The letters on the X axis demonstrate their leukotriene C_4_ synthase promoter polymorphism status (A-444C, rs730012).

There was no difference in the change in FEV_1_ between the botanical oil and placebo arms after 6 weeks of treatment in the study cohort as a whole, and in the group of A homozygotes. In contrast, in C allele carriers (n = 9), the FEV_1_ improved 3% after treatment with combination botanical oils and declined 4% after placebo corn oil (difference of 7% between drug and placebo, p = 0.02, Figure 3). More importantly, all 9 individuals with the C allele, but only 7 of 19 individuals homozygous for the A allele, showed an improvement in their FEV_1_ when receiving the botanical oil combination compared to placebo (p = 0.007 for the percentage of responders, Figure 4).

**Figure 5:**
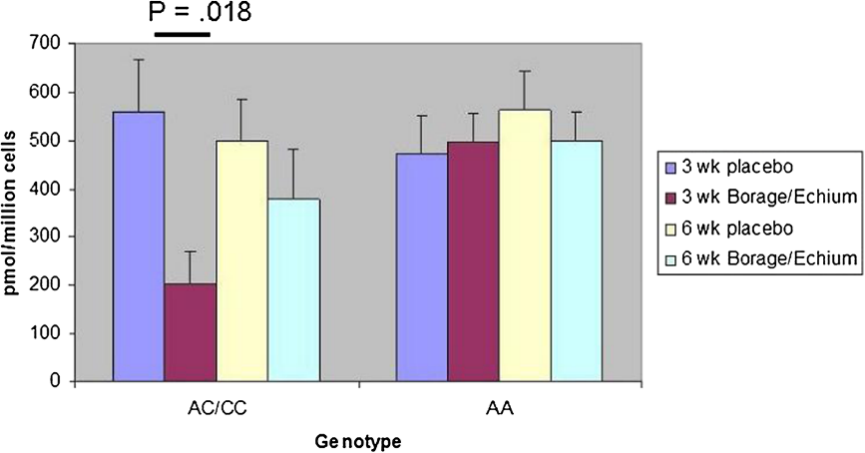
**Effects of dietary supplementation with borage and echium seed oils on 5-LO pathway product formation by ionophore-stimulated peripheral blood granulocyte fractions from stable asthmatic subjects with AA and AC/CC** ***LTC4S*** **genotypes. a**. Sum of total 5-LO pathway products (5-HETE, all-*trans*-LTB_4_, LTB_4_, and cys-LTs) generated per 1 × 10^6^ ionophore-stimulated granulocytes in each indicated genotype after 3 and 6 weeks of treatment on each arm. **b**. Non-cys LTs (5-HETE, all-*trans-*LTB_4_, LTB_4_) **c**. cys-LTs (LTC_4_, LTD_4_, and LTE_4_) were measured for the same samples.

Baseline urinary LTE_4_ levels did not differ between the genotypes and showed no changes in either group on either arm of the study (data not shown). The two genotype groups showed similar levels of LTs generated by basophils (with FcϵRI cross-linkage) and granulocytes (with ionophore stimulation) before randomization. However, generation of ionophore-induced 5-lipoxygenase pathway products was markedly reduced during treatment with the botanical oil combination in the C allele carriers, but not in the A allele homozygotes. This difference was substantial at the first measurement after 3 weeks of therapy (Figure 5a), but lost significance after six weeks. The majority of the products detected were LTB_4_ and proximal 5-LO pathway products (5-HETE and all-trans LTB_4_ (Figure 5b). CysLT production as a fraction of the total did not vary by genotype, and trend of CysLT generation tended to follow the overall trend for 5-LO production, but the difference between the treatment arms in the C allele carriers after 3 weeks of treatment did not reach statistical significance (p = 0.06, Figure 5c).

Overall, the treatment was well tolerated. We did not observe liver dysfunction or a significant drop in hemoglobin concentrations, which have been reported with botanical seed oil consumption.

## Discussion

This study demonstrates that a combination of omega-3 and omega-6 dietary medium chain PUFAs (containing SDA and GLA, respectively), provided over six weeks in a fixed ratio in the form of borage and echium seed oils, results in a significant difference in FEV_1_ compared with corn oil placebo in subjects bearing at least one copy of a polymorphic LTC_4_S allele in a small proof-of-concept crossover study. This is the first study to suggest that botanical oil supplementation can modify lung function and thus have therapeutic potential in asthma if used in a personalized manner.

Dietary supplementation with omega-3 PUFAs from fish oil significantly reduces exercise-induced bronchoconstriction as well as concentrations of CysLTs and prostaglandins in induced sputum in elite athletes (Mickleborough et al. 2006; Tecklenburg-Lund et al. 2010). Omega-3 PUFAS were equivalent to the effects of the CysLT1 receptor antagonist montelukast on blocking bronchoconstriction caused by eucapnic hyperpnea in the latter study (Tecklenburg-Lund et al. 2010). Nagakura et al. (2000) found that dietary supplementation with fish oil rich in omega-3 PUFAs EPA and DHA improves symptoms and airway hyper-responsiveness in children with asthma living in a health care facility with strictly controlled environment in terms of inhalant allergens and diet. In a random sample of adults in The Netherlands studied between 1994 and 1997, McKeever et al. (2008) found that a high intake of omega-3 fatty acids does not protect against asthma, but a high intake of several omega-6 fatty acids is associated with a significant reduction in FEV_1._ They proposed that the high dietary intake of omega-6 fatty acids, rather than reduced omega-3 intake, may have an adverse effect on lung health. There is a possible detrimental effect of oil and fat consumption in asthma. Wood et al. (2011) examined the effect of a single high-fat meal versus low-fat meal on the bronchodilator response to albuterol in asthmatics. The high fat meal contained 48 g (49% of total energy) total fat, including 20.5 g (21% of total energy) saturated fat. In our study, we did not control the diets our subjects consumed while in the study; hence it is likely they were all are eating a typical modern Western diet which contains about 125 g of fat/day. Our protocol provided 9 g of fat given throughout the day and only a small proportion (~2.5 g) of that were the proposed bio-actives (GLA and SDA). Consequently it is unlikely that providing this small amount of fat on top of such a background diet would have had any impact on the clinical and other parameters examined, based on the mass of the fat alone.

5-lipoxygenase (5-LO)-dependent oxidative metabolism of arachidonic acid (AA) leads to generation of pro-inflammatory leukotrienes (LTs). However, dihomo-gamma-linoleic acid (DGLA), a precursor of AA, suppresses LT generation by competing with AA for substrate utilization by 5-LO. Borage (*Borago officinalis*) seed oil is a rich source of gamma linolenic acid (GLA), which is the precursor of DGLA (Figure 1). Dietary supplementation with Borage seed oil provides effective inhibition of leukotriene generation as demonstrated by significant attenuation of LT biosynthesis from circulating granulocytes *in vitro* in cell culture and *ex vivo* (Henz et al. 1999; Johnson et al. 1997; Ziboh and Fletcher 1992). However, hepatic metabolism of GLA to AA by ∆-5 desaturase increases circulating free AA levels, which has the potential of neutralizing DGLA’s potential as a leukotriene synthesis modifier (Ferretti et al. 1997; Johnson et al. 1997; Kelley et al. 1997; Seyberth et al. 1975). Supplementation of diet with long chain omega-3 fatty acids such as eicosapentaenoic acid (EPA) found in fish oils have been shown to compete with DGLA at the ∆-5 desaturase step preventing elevations in AA as a result of providing GLA in borage oil (Barham et al. 2000; Surette et al. 2008). In the current study, the botanical omega-3 PUFA, stearidonic acid (SDA) from the seed oil of echium (*Echium plantagineum*) was utilized because it bypasses the rate-limiting ∆-6 desaturase in long chain, omega-3 PUFA biosynthesis and thus is efficiently converted to EPA.

Because the effects of botanical omega-3 and omega-6 PUFAs on asthma had not previously been studied, we sought to determine whether borage and echium oils provided in the ratio defined in our preliminary dose-titration study could impact FEV_1_ when compared with a placebo in a small, crossover study of stable asthmatics (Arm et al. 2013). In our study, none of the secondary outcomes, including asthma symptoms, rescue inhaler use and peak expiratory flow, improved either in the cohort as a whole or in either genotype group. This is likely because the majority of participants enrolled in the study were relatively well-controlled and the study was likely underpowered to identify these differences.

It is well known that FEV_1_ measurements show substantial heterogeneity in response to treatments, including to 5-LO inhibitors (Drazen et al. 1999), CysLT1 receptor antagonists (Tantisira and Drazen 2009), and even the gold standard therapy of inhaled glucocorticoids (Tantisira et al. 2011). Since we anticipated that any therapeutic benefit of the botanical omega-3 and omega-6 lipids would reflect a modulatory effect on LT generation, we performed a pre-specified analysis of genotyping at the LTC_4_S locus for the common A to C variant that has been associated with numerous outcomes reflective of altered CysLT generation (Asano et al. 2002; Acevedo et al. 2007; Sayers et al. 2003). We observed two distinct effects of the variant C allele on the response to the botanical oil supplementation. First, the C allele carriers showed a net difference of 7% in FEV_1_ between the placebo corn oil and botanical oil arms (Figure 3), and all 9 of them demonstrated their highest FEV_1_ measurements while on the study botanical combination (Figure 4). In contrast, the A allele homozygotes showed no change in FEV_1_ at any time during the course of the study. The 7% difference in FEV_1_ is comparable to the efficacy of montelukast seen in patients with mild persistent asthma with near-normal lung function (Barnes et al. 2001).

Second, the granulocytes from the C allele carriers, but not those from the A allele homozygotes, showed a transient but significant, reduction in overall 5-LO product formation in response to ionophore (Figure 5). In contrast to our preliminary dose-titration study, in which botanical oil effects on LT formation by granulocytes and basophils were compared to pre-treatment levels, the second study involved a corn oil placebo. Thus, we cannot exclude the possibility that the corn oil (containing relatively high levels of the omega-6 PUFA, linoleic acid) could have altered LT generation so as to modify the evident effect of the botanicals. Indeed, this seemed to be the case for FEV_1_, in which the difference was due to both an increase in FEV_1_ with the botanical oils and a decrease in FEV_1_ with the corn oil – an observation that was restricted to the C allele carriers (Figure 5a). Since the effect of the botanicals was significant for total 5-LO pathway products and for non-CysLTs (Figure 5), we suspect that the variant LTC_4_S allele may alter the response of the 5-LO pathway to dietary omega-3 and omega-6 PUFAs in a manner independent of its primary function of conjugating LTA_4_ to glutathione, perhaps from an epistatic effect.

In summary, we conclude that as compared to corn oil, a combination of borage and echium seed oils containing 1.7 g/day of γ-linolenic acid and 0.8 g/day of stearidonic acid improves airflow obstruction in mild to moderate asthmatics who carry the variant allele in the LTC_4_ synthase gene promoter (A-444C). Our findings suggest that botanical oil supplementation can have therapeutic potential in asthma if used in a personalized manner. Further studies examining the effects of botanical oil combinations in asthma are warranted.

## Methods

**Figure 6:**
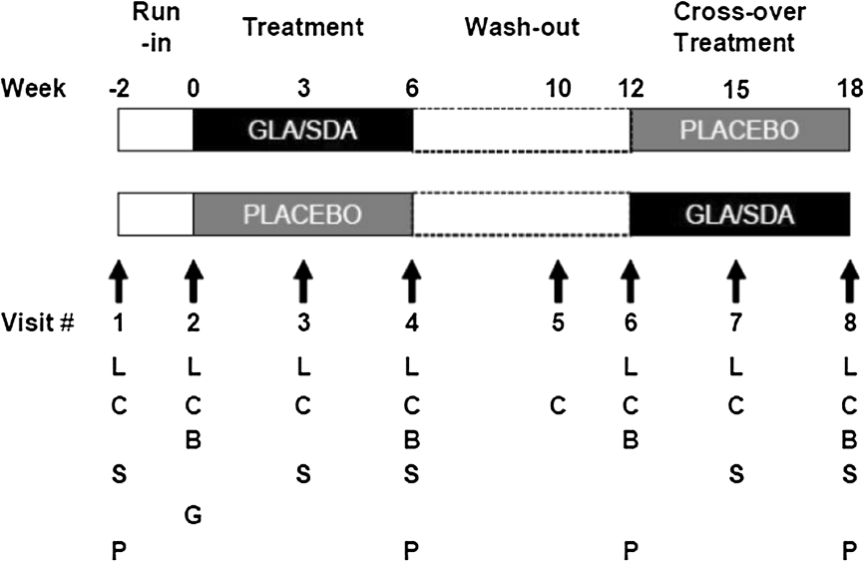
**Study design.** Illustrates study visits and periods of run-in, treatment, and wash-out. GLA = gamma-linolenic acid; SDA = stearidonic acid; Placebo = corn oil; B = blood draw for measurement of biochemical outcomes; C = provision and review of diary cards; G = blood draw for genomic analysis; L = measurement of lung function by spirometry; P = pregnancy test; S = safety monitoring.

We designed a randomized, double-blind, placebo-controlled, cross-over clinical trial (Figure 6). The protocol was approved by the Partners Human Subjects Research Committee. An investigator-initiated IND was obtained from the US Food and Drug Administration (IND number 74,110). Mild to moderate asthmatics with a physician diagnosed history of asthma and the presence of variable airflow obstruction were recruited. Patients on leukotriene modifiers, oral or high dose inhaled steroids, theophylline or omalizumab (a monoclonal antibody against Immunoglobulin E) were excluded. Medical and respiratory history, a brief physical examination and routine laboratory tests were conducted at the screening visit to exclude the presence of significant co-morbid diseases.

The study design is depicted in Figure 6. The primary outcome was pre-specified to be the difference in the change in forced expiratory volume in one second (FEV_1_) after three weeks of therapy between combination botanical oil and placebo therapy. Spirometry was performed with the KoKo Spirometry software. Pre-specified secondary analyses included the examination of the effect of A-444C polymorphism of the LTC_4_S gene on FEV_1_, rescue inhaler use, daily symptoms documented in diary cards, daily lung function assessed with Jaeger electronic peak flow meters, leukotriene generation from peripheral white blood cells in response to a range of stimulus doses and urine LTE_4_ measurements. Details of assays used for measurements are provided in the online resource accompanying the manuscript. Blood from participants was collected weekly for assessment of hematological indices, liver and renal function. Medication compliance was monitored by medication diaries, counts of returned capsules, and plasma fatty acids measurements.

Capsules containing a combination of borage and echium seed oils were obtained from Croda Europe Ltd (Leek, Staffordshire, UK). Subjects randomized to receive drug were given nine 1 g capsules (7 with echium and 2 with borage oil) each day, for a total of 1.57 g and 0.87 g gamma-linolenic acid (GLA) and stearidonic acid (SDA), respectively, per day. Subjects randomized to placebo received 9 capsules of corn oil each day. Each 1 gram capsule of corn oil contained no GLA or SDA and 560 mg of linoleic acid (the major fatty acid in corn oil) for a total dosage of 5 g of linoleic acid. The typical Western diet contains very high levels of linoleic acid (6-8% of daily energy) so this supplementation protocol would add a very small percentage of the linoleic acid found in the typical modern Western diet.

We used Student’s t-tests to compare normally distributed data, Wilcoxon rank sum tests for unpaired non-parametric data and Wilcoxon signed rank tests for paired non-parametric data. Categorical data were analyzed using two-sided Fisher’s exact tests. We employed repeated measures mixed model analysis in which repeated measures were taken on each subject at each visit (time) and the group and time interactions were modeled with the main effects. A compound symmetry correlation structure was used since the correlation between pairs of times would be similar and not expected to change across time. Data were analyzed using SAS 9.1 (SAS Institute Inc., Cary, NC, USA) where the significance level is set at 95%. All subjects were genotyped for their LTC_4_S A-444C polymorphism status at the Harvard Partners Center for Genetics and Genomics (HPCGG) by using either Sequenom MassARRAY system (Sequenom, San Diego, CA) or Taqman analysis on the Applied Biosystems 7900HT system (Applied Biosystems, Forster City, California, USA) based on assay availability in the laboratory. Quality control was assured by running internal and external controls on all genotyping plates.

## Abbreviations

5-HETE: 5 hydroxyeicosatetraenoic acid
5-LO: 5 lipoxygenase
AA: Arachidonic acid
CysLT: Cysteinyl leukotriene
DGLA: Dihomo γ-linolenic acid
DHA: Docosahexanoic acid
DPA: Docosapentaenoic acid
EPA: Eicosapentoenoic acid
FcϵR1: Fc receptor for IgE
FEV_1_: Forced expiratory volume in one second
FADS: Fatty acid desaturase
GLA: Gamma-linolenic acid
LA: Linoleic acid
LT: Leukotriene
LTC_4_S: Leukotriene C_4_ synthase
LTE_4_: Leukotriene E_4_
PUFA: Polyunsaturated fatty acid
SDA: Stearidonic acid.
